# Rapid conversion of an in-patient hospital unit to accommodate COVID-19: An interdisciplinary human factors, ethnography, and infection prevention and control approach

**DOI:** 10.1371/journal.pone.0245212

**Published:** 2021-01-22

**Authors:** Raad Fadaak, Jan M. Davies, Marlot Johanna Blaak, John Conly, Joanne Haslock, Ashley Kenny, Nicole Pinto, Myles Leslie

**Affiliations:** 1 School of Public Policy, University of Calgary, Calgary, Alberta, Canada; 2 W21C Research and Innovation Centre, University of Calgary, Calgary, Alberta, Canada; 3 Department of Anesthesiology, Perioperative and Pain Medicine—Cumming School of Medicine, University of Calgary, Calgary, Alberta, Canada; 4 Departments of Medicine, Microbiology, Immunology and Infectious Diseases—Cumming School of Medicine, University of Calgary, Calgary, Alberta, Canada; 5 Snyder Institute for Chronic Diseases, University of Calgary, Calgary, Alberta, Canada; 6 O’Brien Institute for Public Health, University of Calgary, Calgary, Alberta, Canada; 7 Infection Prevention and Control, Alberta Health Services, Calgary, Alberta, Canada; 8 Foothills Medical Centre, Alberta Health Services, Calgary, Alberta, Canada; 9 Department of Community Health Sciences—Cumming School of Medicine, University of Calgary, Calgary, Alberta, Canada; Thomas Jefferson University, UNITED STATES

## Abstract

**Background:**

In response to the Coronavirus disease-19 (COVID-19) pandemic, in-patient units in hospitals around the world have altered their patient care routines and Infection Prevention and Control (IPC) practices. Our interdisciplinary team of applied Human Factors (HF), ethnography, and IPC experts assisted one Unit, normally serving general surgical and orthopedic patients, as it rapidly converted to deliver COVID-19-specific care. This paper describes the conversion experience of the Unit, and outlines broader lessons for other acute care teams faced with similar issues.

**Methods:**

We deployed walkthroughs, simulations, and ethnography to identify important safety gaps in care delivery processes on the Unit. These interventions were undertaken using interdisciplinary theories of implementation that combined systems-level HF perspectives, ethnographic approaches, and individual-level IPC perspectives. Timely recommendations were developed and delivered to Unit staff for feedback and implementation.

**Results:**

We describe three interventions on the Unit: 1) the de-cluttering and re-organization of personal protective equipment (PPE); 2) the reconfiguring of designated ‘dirty’ tray tables and supplies; and 3) the redesign of handling pathways for ‘dirty’ linens and laundry. Each of these interventions was implemented to varying degrees, but all contributed to discussions of safety and IPC implementation that extended beyond the Unit and into the operations of the broader hospital.

**Conclusions:**

Leveraging our team’s interdisciplinary expertise and blended approaches to implementation, the interventions assisted in the Unit’s rapid conversion towards providing COVID-19-specific care. The deployment and implementation of the interventions highlight the potential of collaboration between HF, ethnography, and IPC experts to support frontline healthcare delivery under pandemic conditions in an effort to minimize nosocomial transmission potential in the acute healthcare setting.

## Introduction

The global spread of Severe Acute Respiratory Syndrome Coronavirus 2 (SARS-CoV-2), and its clinical manifestation as Coronavirus Disease 2019 (COVID-19), has resulted in immense upheaval and strain across healthcare systems [1,2]. The widespread closures of health services, cancellations of elective procedures [1], and the rapid depletion of hospital resources in the initial wave of the pandemic [3] have forced acute care staff and clinicians to quickly redesign structures, processes and procedures to adapt to the pandemic.

An emerging body of literature illustrates how individual hospitals and units redesigned their overall operations during the first wave of the pandemic [4–14]. In this paper we describe: 1) how a general surgical and orthopedic unit in a tertiary, 1100-bed general hospital in Calgary, Alberta, Canada was rapidly repurposed to become a COVID-19 care unit, and 2) the role played by an expert interdisciplinary team in the Unit’s rapid conversion.

We focus on three specific interventions to illustrate the collaborative potential inherent in combining the expertise and implementation theories of applied Human Factors (HF), ethnographic, and Infection Prevention and Control (IPC) professionals. The three interventions were developed by our interdisciplinary team working with Unit management and staff. The specific composition, as well as the broader theoretical foundations and deployment of the HF, ethnography, and IPC team have been described elsewhere [15]. Here we describe the interventions in detail, as well as examine the implementation theories that our interdisciplinary team blended to deliver support as the Unit rapidly converted to COVID-19-specific care.

## Methods

### Context

Unit 64 is situated within the Foothills Medical Centre (FMC), the largest hospital in the province of Alberta, Canada. Like all hospitals in the province, FMC falls under the jurisdiction of Alberta Health Services (AHS). Formed in 2008 through the amalgamation of nine regional health entities to create a centralized health system, AHS is the sole health authority in the province of Alberta and is publicly-funded. It functions as the operational arm of the Ministry of Health (‘Alberta Health’), and is responsible for delivering care in a spectrum of facilities that is predominated by acute care hospitals, but includes public health centres, mental health clinics, and continuing care sites.

Unit 64 is a 40-bed hospital unit (10 single rooms, 13 two-bed rooms, one four-bed room) that normally serves FMC’s general surgical and orthopedic inpatients. It is located in the hospital’s newer (2010) McCaig Tower. On 23 March 2020, 18 days after Alberta reported its first presumptive case of COVID-19, Unit 64 was designated by FMC administration as the hospital’s primary treatment destination for COVID-19 patients, a change which was to occur within 24 hours. As a result, 23 existing patients were transferred off the Unit or discharged between 13h00 and 22h00, and by the end of the day, Unit 64 began receiving COVID-19 patients. The rapidity of this conversion from surgical and orthopedic care delivery to a COVID-19-focused Unit delivering medical care to respiratory patients required significant changes to routines and protocols for nursing, housekeeping, and healthcare aides, as well as porters and medical staff.

### Engagement

Our team of applied HF and ethnography researchers initiated contact with Unit 64 on 3 April 2020. The HF and ethnography team was composed of two HF experts (MJB, JMD), and two ethnography experts: a medical anthropologist (RF), and a medical sociologist (ML). Our contact with the Unit was made through the FMC’s IPC leadership (JC), who had previously engaged with hospital administration to assist in converting the Unit for COVID-19 care. Our initial mandate was to support Unit 64 and work in concert with IPC to provide discrete recommendations to improve the layout, placement, and use of Personal Protective Equipment (PPE) and other safety supports. Throughout subsequent consultative sessions, our interdisciplinary team continued to engage with Unit 64 managers, the Clinical Nurse Educator, nursing staff, housekeepers and housekeeping managers on the Unit.

The collaboration between Unit leadership, IPC staff, and the HF and ethnography team members continued through June, July, and August 2020, even after surgical and orthopedic services resumed alongside COVID-19-specific care. This ongoing work aided Unit 64 staff to revert to caring for post-operative patients, while continuing to enact the COVID-19 care practices and routines that had become their ‘new normal’.

### Data collection and analysis

For this study, our HF and ethnography team members used cognitive walkthroughs [16], rapid task analyses [17,18], and ethnographic observations to collect data. Our cognitive walkthroughs were conducted by our team, first with the team alone and then together with Unit leadership. All elements of data collection were done on the Unit floor, in real-time, with feedback provided immediately to Unit management and staff. This was due to the urgency related to the pandemic and the recent transformation of the Unit.

Data collection involved direct observations of spatial configuration, the placement of supplies, staff protocols, and tasks and interactions. Data collection focused on identifying systemic factors on the unit, including IPC issues related to work routines and staff tasks. Each walkthrough lasted approximately 45 minutes, including the team’s observation period, followed by real-time feedback and discussion of the observed issues with Unit leadership and staff. Seven walkthroughs were conducted between April and August 2020, for a total of nearly 7 hours. Ethnographic observations (n = 7) were conducted as a follow-up to each of the walkthroughs. This included direct on-site ‘shadowing’ sessions, using an exploratory approach where observations were combined with on-the-spot, unstructured interviews with Unit staff. These ethnographic ‘shadowing’ sessions aimed to validate the observations from the walkthroughs, and to focus on how our team’s recommended changes were, or were not, implemented on the Unit.

Data collection and analysis were done concurrently. Analysis of information from the walkthroughs involved the discussion and categorization of observations and key areas of intervention between our team (JD, JB, RF) and Unit leadership (JH, AK). Emerging themes were drawn from ethnographic observations and interviews (RF) and used to structure and update the recommendations provided to the Unit. Data collection, processing and analysis from both the walkthroughs and ethnographic observations were discussed regularly between our HF and ethnography teams, as well as Unit leadership.

### Interdisciplinary theories of change

Our recommendations emerged from a blending of implementation theories, which are assumptions and rationales about system features and human actions that are required to promote the translation of objectives into effective program and service delivery [19,20]. Our HF team members brought a ‘systems-level’ theory of implementation to the task of converting Unit 64. In HF, a system is a holistic concept defined as an assemblage of constituents that interact to fulfill a common purpose [21]. In this regard, our applied HF experts were focused not on policy documents, but the broader mix of people, objects, and physical spaces in which Unit 64 staff sought to interpret and implement the directives provided in policy documents. Along with Unit leadership, our HF team members focused on systems rather than individual attitudes or behaviours to determine how these were supporting, or undermining safety. Here we diverged from a ‘person-centric model’ of evaluation, and instead deployed a theory of change that targeted individual users as embedded in the systems that surround those users [22].

Similarly, our applied ethnographic researchers were focused on social and institutional contexts as key systems-level factors shaping the implementation of recommendations. We worked first as credible outsiders and became trusted ‘alongsiders’ [19,23], able to provide non-binding recommendations to the staff based on our mutual observations and co-designed solutions [24]. Whether or not these recommendations were implemented was entirely contingent on both IPC validation and Unit staff acceptance.

Finally, our IPC professionals combined attention to systems-level factors with individual-level theories of implementation. By blending this perspective, our team did not entirely abandon the ‘person centric’ approach to evaluation [22], but maintained a flexibility to targeted individual users, as well as the systems that surround those users [25,26]. Combining these three approaches to achieving implementation success–systems, ‘alongsider’, and individual–was a key feature of the interdisciplinary team’s contribution.

The following sections describe three interventions that we have selected as particularly illustrative of our team’s contributions to Unit 64’s conversion. The first section focuses on an initial intervention to declutter and improve PPE organization and layout on the Unit; the second focuses on the promotion of improved hygienic practices for tray tables that were used to house supplies outside patients’ rooms; and the third examines co-designed efforts to streamline processes for managing dirty linens with Unit management, IPC, and housekeeping staff.

### Reporting

Our team followed the Standards for Reporting Qualitative Research (SRQR) [27] to report on the findings from this intervention.

### Ethics

Ethics approval for the full study was granted by the University of Calgary’s Conjoint Health Research Ethics Board (CHREB) on 11 March 2020 (REB20-0371).

## Results

### De-Cluttering and improving organization of PPE

#### Context on the unit

We first deployed our ethnographer who took observations of the floor layout and staff practices. Our HF team members then organized an initial walkthrough of the Unit, which was shortly followed by a second walkthrough with the Manager, Clinical Nurse Educator and the ethnographer.

**Fig 1:**
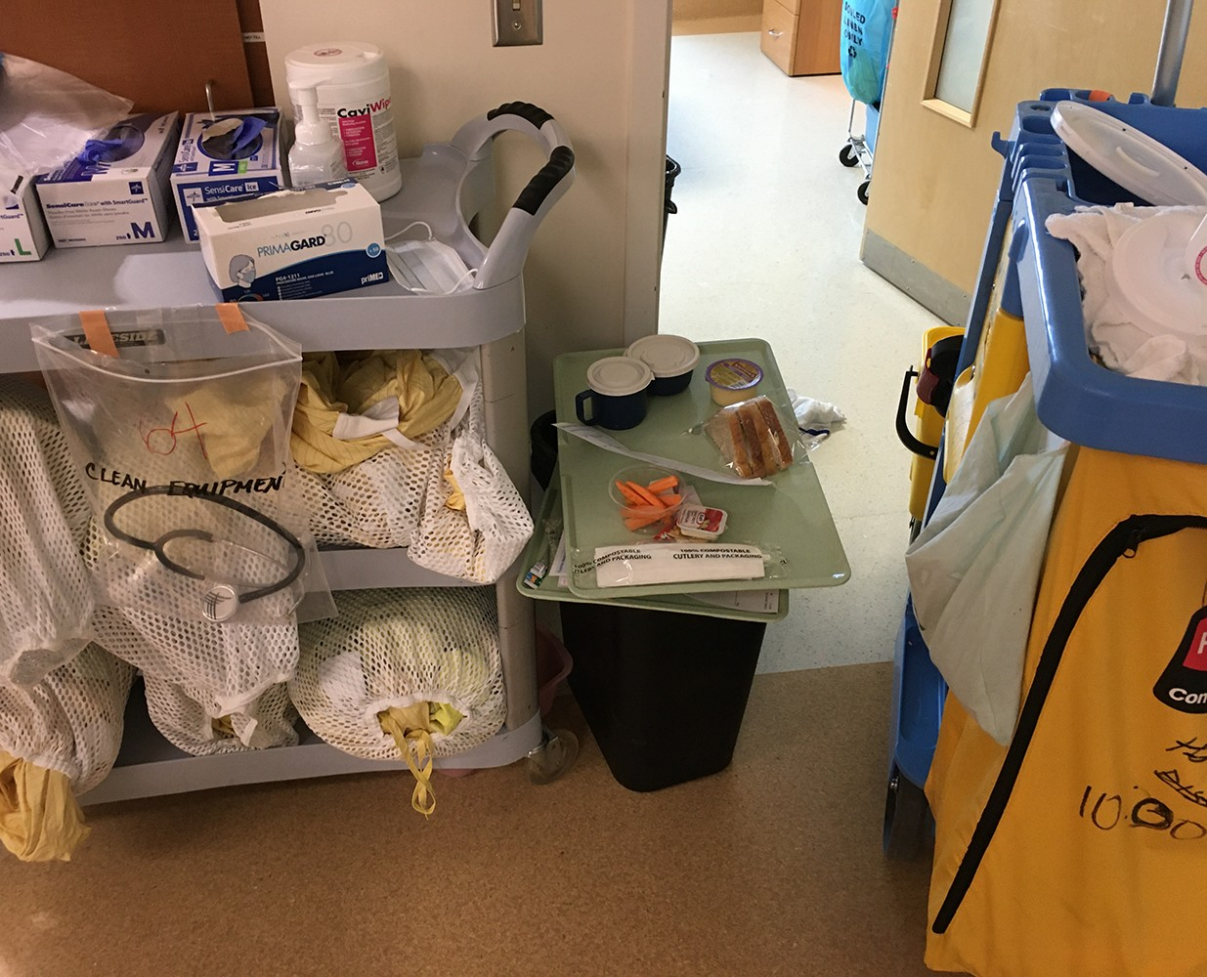
HF/ethnography team members observed clean PPE (gowns, gloves, and supplies) stored haphazardly on chairs, carts, and tray tables throughout the hallways of the unit during our first walkthrough. Note food tray awaiting delivery placed on waste bin.

**Fig 2:**
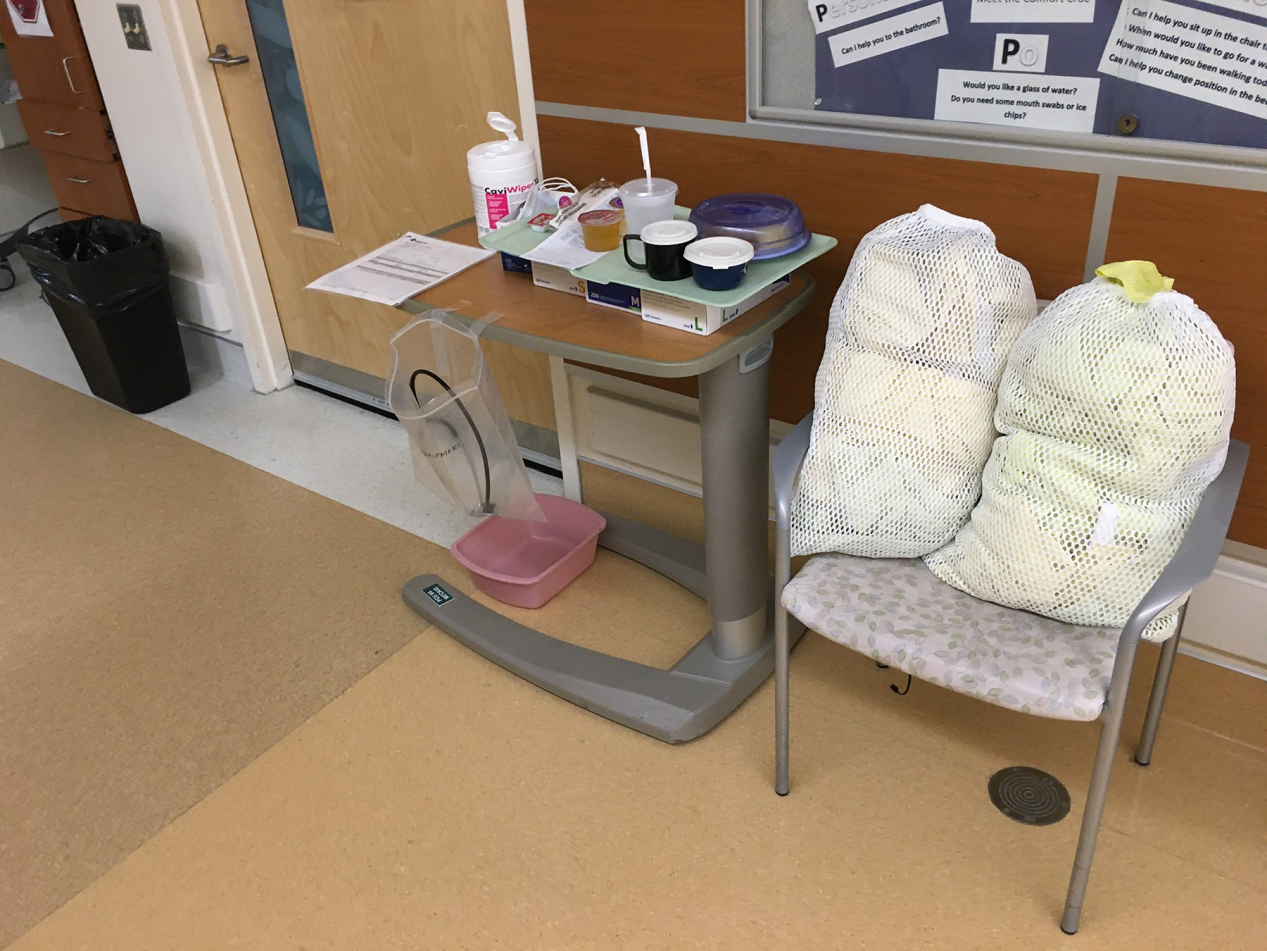
Clean and contaminated items were stored in close proximity to one another. There was no clear indication if the plastic bag was dedicated for clean or contaminated stethoscopes. The pink basin located on the floor was intended for dirty equipment, which was placed too close to clean PPE and was not positioned with the black waste bin across the doorway.

During the initial walkthrough, our HF experts made safety-critical observations about the placement of PPE for donning and doffing. The hallways were cluttered with PPE supplies, with clean gowns kept in bundles on chairs in charting areas (Fig 1). Additionally, supplies for donning and for doffing were often in close proximity, rather than being separated by two metres. These supplies were kept on re-purposed patients’ bedside tables, placed in the hallways outside patients’ rooms, which added to overcrowding (Fig 2).

Based on these observations, the interdisciplinary team discussed with IPC and met with Unit leadership to provide safety recommendations and co-designed solutions. Our initial recommendations and solutions were immediately presented by the Manager to the attending unit staff in a physically-distanced huddle at the Nursing Station.

#### Recommendations delivered to the unit

The following recommendations were shared with the Manager and Clinical Nurse Educator:

- Ensure separation of clean PPE from doffing spaces and contaminated items. Consolidate and simplify the PPE stations by positioning one donning cart between two single patient rooms.
- Provide additional doffing garbage and ‘dirty’ linen hampers inside individual rooms to minimize clinicians walking while wearing contaminated PPE to reach these bins. Ensure these bins are located to match the expected orientation (i.e., left versus right) for the clinician as he/she exits a patient’s room to doff remaining contaminated PPE (see [28] for AHS’ standard PPE doffing protocols).
- Ensure that dedicated ‘clean’ stethoscopes are made available in trays positioned on donning carts outside the rooms. Contaminated stethoscopes should be put into ‘dirty’ bins upon exiting patients’ rooms and double-wipe disinfected before being returned to the clean trays.
- Post AHS-approved PPE doffing checklists on the inside of patients’ rooms so that they are clearly visible to staff for reference while doffing before exiting.

#### Implementation on the unit

A number of these recommendations were provided in-person at the time of the walkthrough to the nursing and clinical staff present at the Nursing Station huddle. HF and ethnography members of the team drafted a formalized and expanded version of the recommendations, with review by IPC members before submission to Unit leadership for consideration.

**Fig 3:**
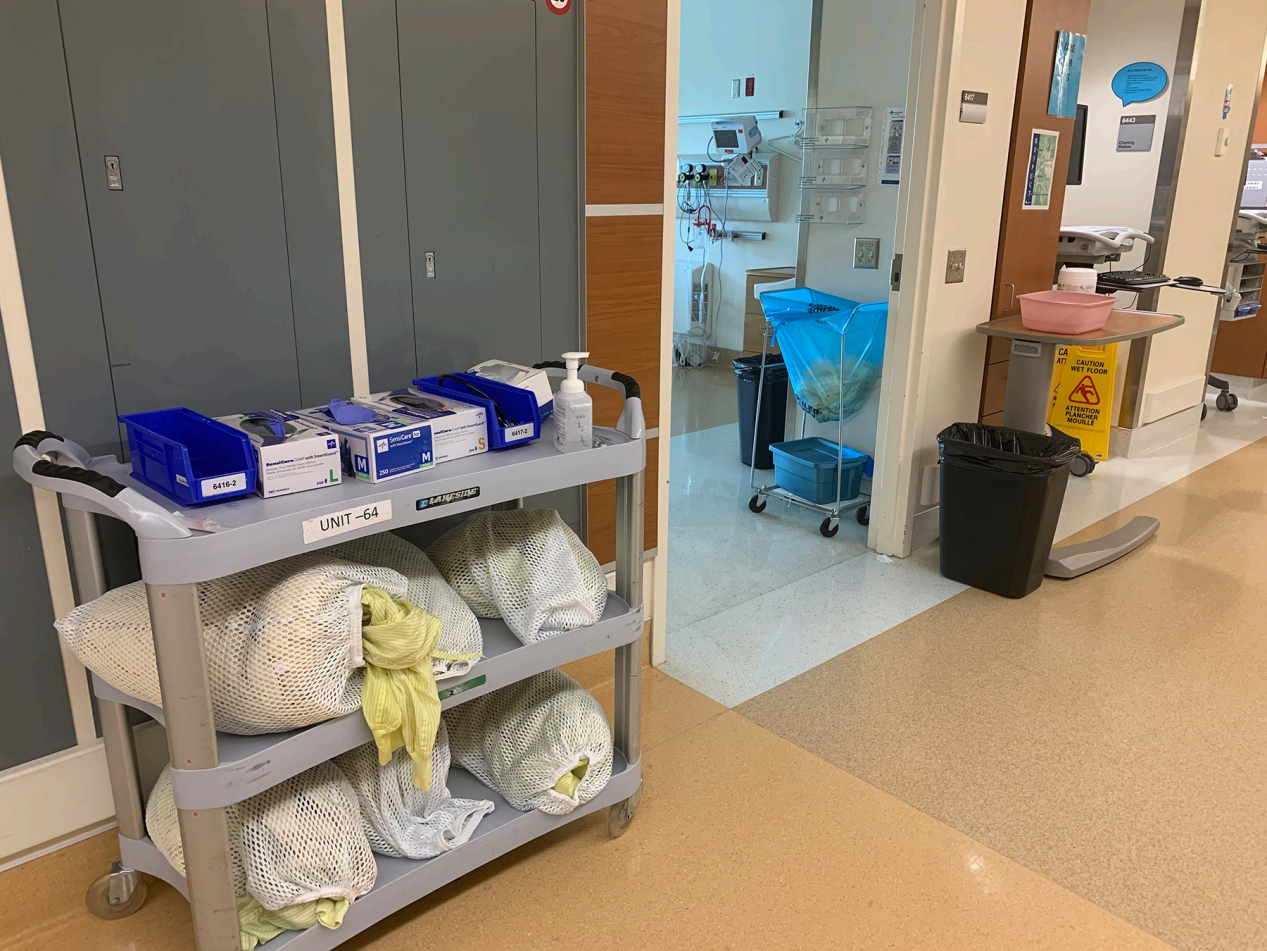
Working alongside unit staff, the team’s recommendations were implemented within 48 hours. Clean PPE was consolidated onto a single cart, with designated areas for gowns, gloves, and clean stethoscopes. Contaminated equipment was confined to a single area outside patients’ rooms, with the pink basin (for dirty goggles and stethoscopes) moved from the floor onto the designated dirty tray table. Doffing bins for gowns and linens were made available inside each room on the same side as the dirty tray tables outside the room.

With the support of Unit 64’s Manager, Clinical Nurse Educator and Unit staff, our recommendations were implemented within 24–48 hours. Our follow-up visit revealed significant safety and layout improvements: clean PPE was consolidated into donning carts that were shared between two rooms and separated from doffing areas; clean gown bags were removed from chairs and tables around charting areas; dedicated bins for clean stethoscopes were placed on the donning carts; and dedicated doffing areas were established outside rooms, using the repurposed bedside tray tables for doffing supplies. These improvements included placement of pink basins for contaminated eye protection and stethoscopes on the tray tables above the waste bins used for doffing gloves or other contaminated supplies (Fig 3). The reorganization of the PPE carts and supplies was based on a principle of simplification, aimed at reducing both cognitive load, as well as reducing contamination risks for clinical staff during donning and doffing.

### Designated dirty tray tables and supplies

#### Context on the unit

Following implementation of our initial PPE recommendations, our HF and ethnographic team members remained engaged with the staff on Unit 64, informally conducting check-ins and walkthroughs. The follow-up visits focused on observing the updated Unit layout and additional changes implemented by the staff for COVID-19-related care delivery. A month after we provided our initial PPE recommendations, our team was actively re-engaged by the Unit leadership and IPC to address potential nosocomial COVID-19 infection risks related to improper use of designated dirty tray tables next to patients’ rooms.

Two additional walkthroughs of the Unit were undertaken: the first was conducted to develop initial recommendations, and the second was held six days later with our HF experts and IPC to validate and adjust these recommendations.

**Fig 4:**
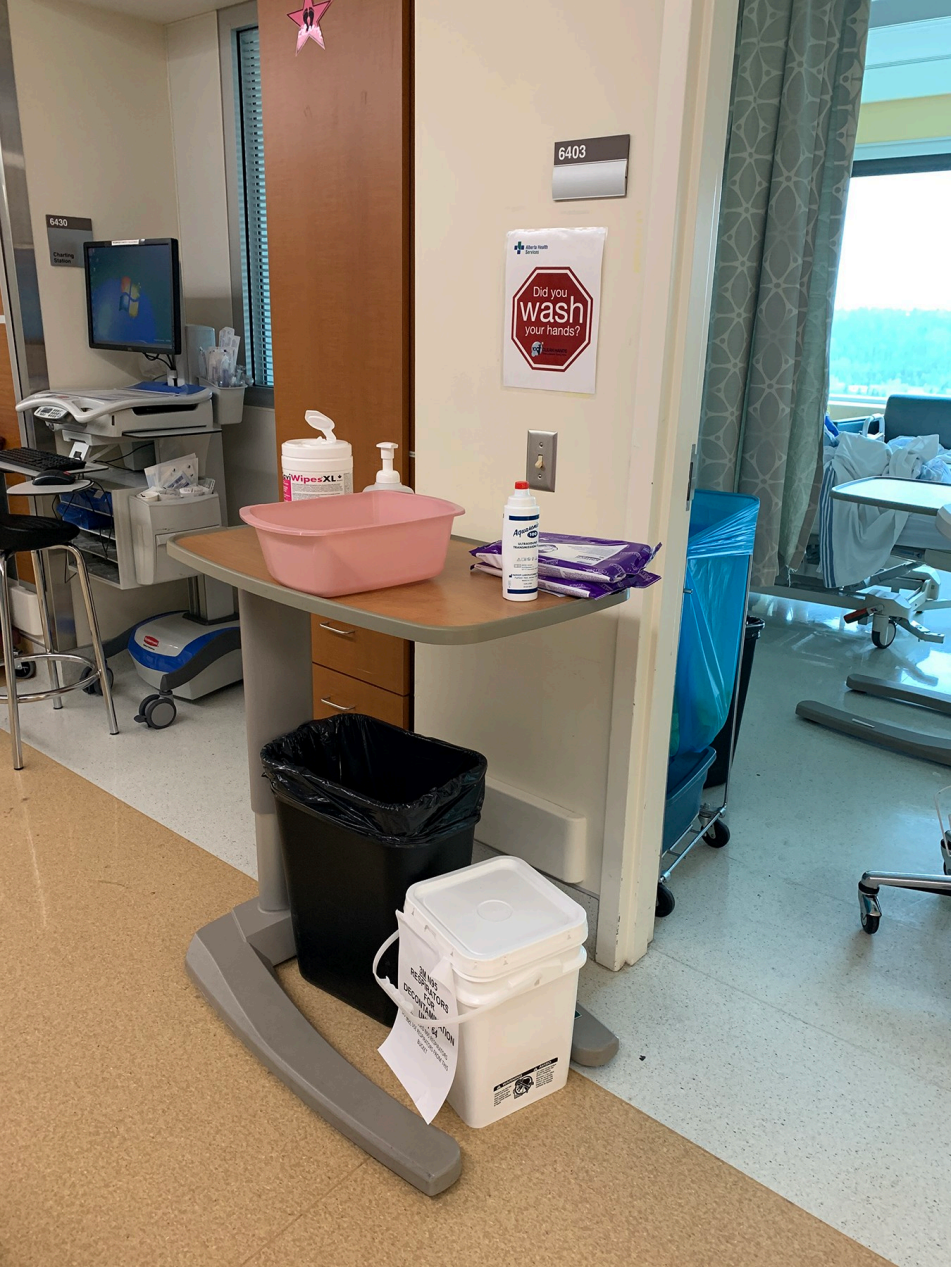
HF/ethnography team members observed clean supplies (pictured here: Sterile wipes and ultrasound gel) being placed on contaminated tray tables outside patients’ rooms.

During our first walkthrough, our HF team observed clinical staff placing clean packs of wipes and ultrasound gel on a designated ‘dirty’ tray table outside a patient’s room, thus contaminating the supplies required for the patient’s examination (Fig 4). Nursing staff and healthcare attendants stated that it was not unusual for the tray tables to be used for temporary placement for both clean items and/or meal trays because they were conveniently located, at ~83 cm, and easily accessible next to room doorways. In addition, because the tray tables were not clearly labeled with ‘dirty’ stickers or colored markings, some staff did not understand that these were designated as contaminated surfaces.

The team recognized that these potential contamination events were driven largely by a structural issue–that designated dirty tray tables offered more convenient placement for clean items and supplies than did the nearby donning carts, which had no spare room for items such as meal trays. Based on this issue, our HF team offered the following recommendations:

### Recommendations delivered to the unit

- Using a red indicator (e.g., tape), clearly mark each tray table with diagonal hatch patterns to indicate the entire space of the designated dirty tray tables are to be considered contaminated.
- Standardizing the placement of the pink basins, hand sanitizer pumps, and Cavi-wipes™ on each tray table, to remind staff to first doff, then hand sanitize, and lastly clean equipment.
- Moving the tray tables and all items close to the doorways to reduce the convenience of the surface for item storage and discourage staff from temporarily placing clean items on the designated dirty trays.

#### Implementation on the unit

Although Unit 64 leadership was willing to accept both these recommendations, IPC team members raised concerns over tape and/or labels being a hindrance for full environmental cleaning. As a result, our team focused on standardizing the placement of the supplies on the ‘designated dirty’ tray tables (79 cm long x 43 cm wide x 84 cm high) to discourage their use for clean supply storage. This placement positioned the pink basins (20 cm long x 23 cm wide)**—**used to hold ‘dirty’ stethoscopes, eye protection, and other reusable small pieces of equipment**—**closest to the doorway entry. The basins were followed by hand sanitizer dispensers (10 cm long x 6 cm wide), then by Cavi-wipe™ dispensers (15 cm diameter). Simply by repositioning the supplies on the tray tables closer to the doorway and reducing unoccupied space to only a few centimetres (~5 cm), these tray tables became much less convenient places for clean items to be temporarily placed or stored. In addition, the order of item placement reflected their order of use, reinforcing proper adherence to doffing protocols. This recommendation was implemented immediately after being shared.

**Fig 5:**
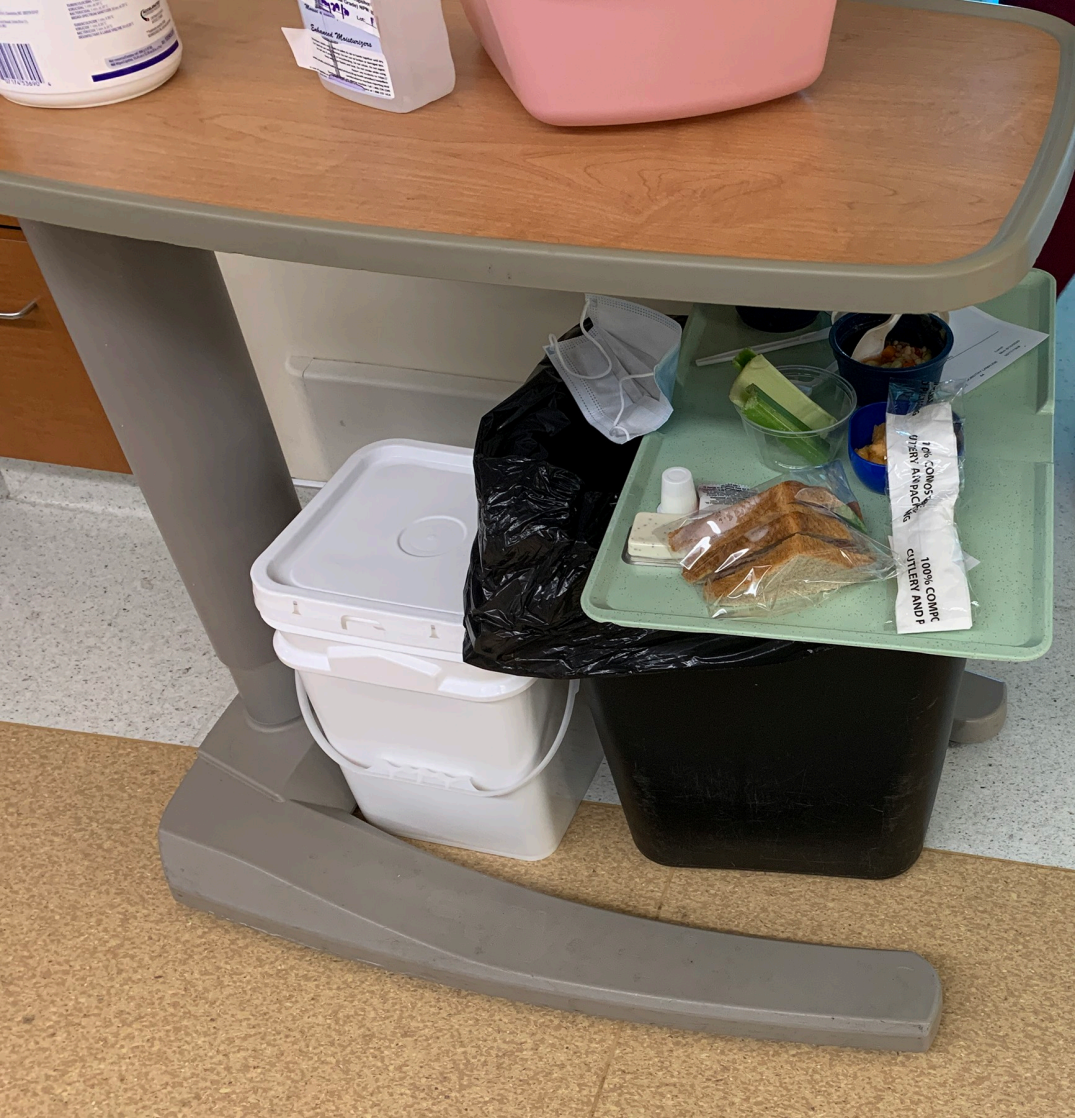
Standardizing the placement of pink basins, hand sanitizer pumps, and Cavi-wipes™ on the ‘designated dirty’ tray tables discouraged their use as a temporary clean supply storage surface. However, clinical staff occasionally ended up placing clean meal trays and other clean equipment on the waste bins beneath the tray tables, as our team discovered during subsequent walkthroughs.

Although standardizing the placement of the supplies housed on the designated dirty tray tables helped hinder their use as temporary storage, clinical staff began to place patient meal trays on top of the waste bins beneath the designated dirty tray tables, as our team discovered on subsequent Unit walkthroughs (Fig 5). This finding was relayed to Unit leadership as an important issue for ongoing vigilance and staff education.

### Dirty linens and laundry pathways re-design

#### Context on the unit

Because of additional concerns about potential contamination events involving dirty linens and laundry, another follow-up visit ensued three months after the Unit was designated for COVID-19 inpatient care. Discussions with IPC and Unit staff revealed that there was no AHS guidance available for the management of dirty linens and laundry. As a result, Unit 64 leadership and some of the staff (nurses and healthcare attendants [HCAs]) had developed improvised solutions to take bags containing dirty linens and laundry from patients’ rooms to be disposed of down the laundry chute. The chute served the entire wing of the hospital and was thus often locked due to active use by other floors/units.

**Fig 6:**
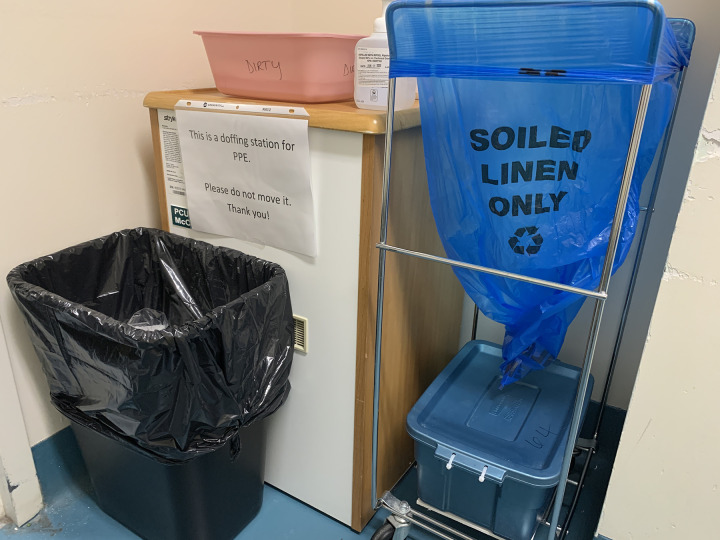
Unit leadership responded quickly to the reports of staff contamination related to dirty linens by installing a dedicated doffing station inside the laundry chute room.

Alongside Unit leadership, HF, ethnography, and IPC team members co-designed solutions to minimize potential staff contamination. We conducted a staff-guided walkthrough, which had us follow and map the journey of dirty linens on the Unit: from a patient’s bedside to an empty linen bag inside the patient’s Isolation Room, down the hall to the Dirty Utility Room and then finally to the Laundry Chute Room. This Laundry Chute Room was located off the Unit, in a small non-clinical wing, where Unit leadership had quickly responded to the reports of staff contamination by installing a PPE doffing station (Fig 6). From the improvised huddle with the assembled teams, further adjustments were made to protocols that would improve, simplify, and increase the safety of the processes involved in the handling of linens and laundry by Unit staff.

Our initial walkthrough was followed up with a subsequent simulation that involved HF, ethnography, IPC, Unit leadership, housekeeping management and housekeeping staff. We initially gathered these stakeholders to reinforce and demonstrate the dirty linen recommendations co-designed during the walkthroughs with IPC and Unit leadership. We also determined how many dirty linens would fill a laundry bag to 50% capacity. This was measured to discourage overfilling of the laundry bags and lower the risk of viral aerosolization, if the contents of the bags were compressed before being tied shut.

During this simulation, we quickly discovered that housekeepers had a pre-existing routine to handle linens on the Unit that differed from the routine used by other Unit staff, such as nurses and HCAs. As such, we also used the simulation as an opportunity to address these differences in task performance, better understand the needs of housekeeping staff, and align the formal process and procedures to ensure that these changes could be adopted by housekeeping staff in different areas of the hospital immediately.

**Fig 7:**
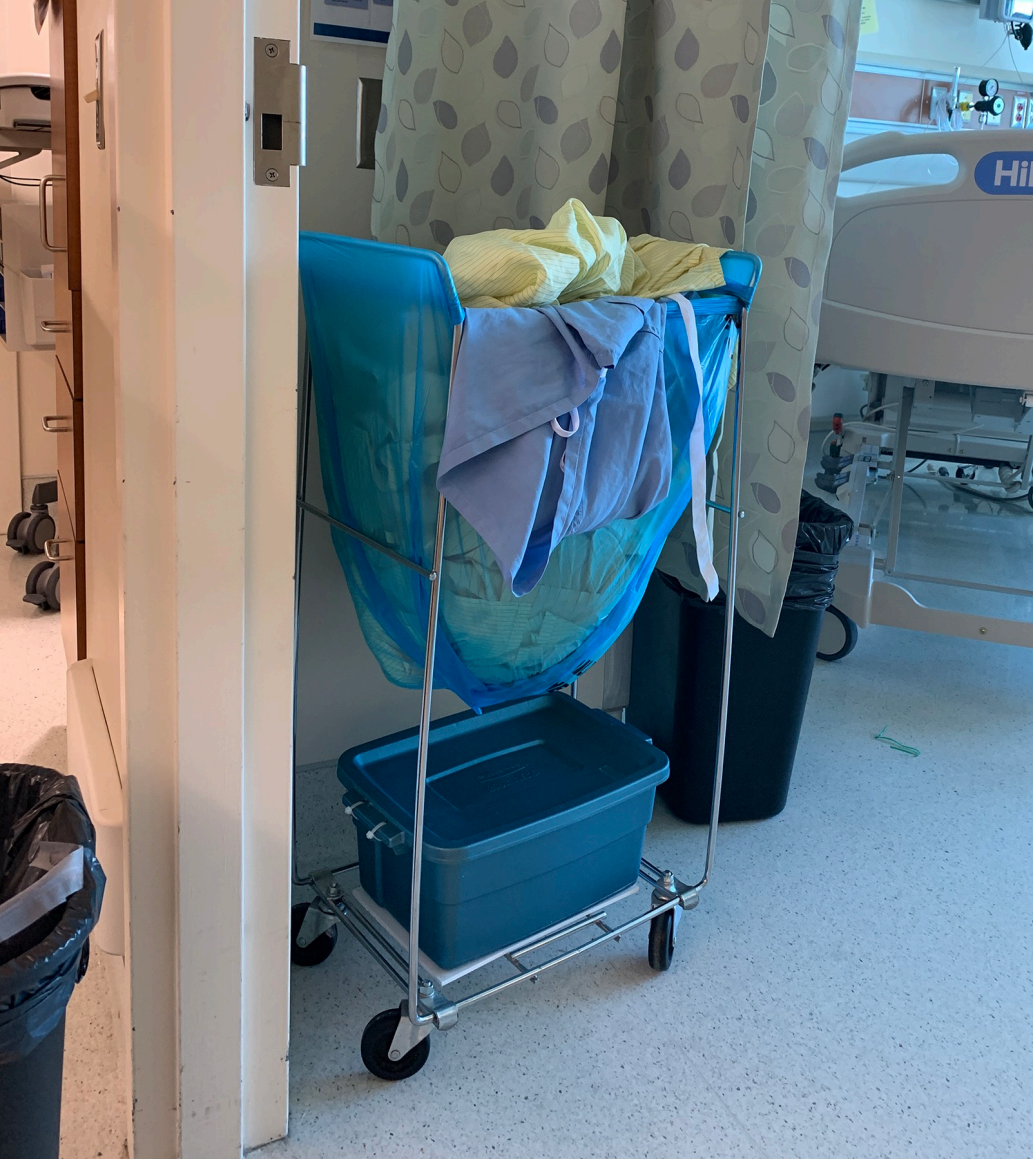
While IPC had assumed that the outsides of linen bags were ‘clean’, during our walkthrough and co-design session we observed that almost every bag had contaminated PPE or linens draped over the sides.

During our initial walkthrough and subsequent co-design huddle, our HF and ethnography team members recognized a key assumption made by IPC and unit staff–that the outside surfaces of the laundry bags were clean–was not aligned with the actual situation on the Unit. In almost all cases, the dirty laundry bags were overflowing, with contaminated sheets and gowns draped over the sides (Fig 7). This observation challenged the recommendation to double- or triple-bag dirty laundry bags from patients’ rooms, as this might increase the risk of healthcare worker and environmental contamination from contact with the outside of the bags. Concerns had also been expressed about the possibility of injuries from lifting heavy bags of dirty laundry when dealing with over-filled bags, especially when double- or triple-bagging.

Recommendations delivered to the unit. Following our initial walkthrough, our HF and ethnography team members recommended:

- Dedicating one of the Unit’s two Dirty Utility Rooms for each half of the Unit, as temporary storage of dirty linen bags.
- Preventing overfilling dirty linen bags (beyond 50% capacity) before replacing them.
- Wearing full Contact and Droplet Precautions PPE (gown, mask, eye protection and gloves) when bagging and transporting linens from patients’ rooms to Dirty Utility Rooms, or from Dirty Utility Rooms to the Laundry Chute Room.
- Preparing clean linen carts before they are required and at the end of each shift, to have an adequate supply ready for replacement in patients’ rooms.

During and following the simulation with housekeeping, our team recommended:

- Placing a second linen bag/frame in each patient’s room to reduce the likelihood of overfilling bags past 50% capacity.
- Doffing dirty PPE prior to exiting patient room. Re-donning clean contact/droplet PPE to transport dirty linen bags from patient room to the dirty utility room or Laundry Chute Room. Doffing dirty PPE after placing bag(s) in the utility room or down the chute.
- Using a permanent marker during adaptation period to show the 50% capacity of empty laundry bags, to assist with visual recognition of appropriate fill levels and to minimize overfilling.
- Using the doffing station in the Laundry Chute Room to minimize the frequency of having staff walk through the Unit’s hallways while wearing contaminated PPE.

#### Implementation on the unit

Shortly following the simulation, the Unit resumed providing post-operative services as well as COVID-19 care. Unit leadership thus decided to designate one Dirty Utility Room for dirty laundry from the surgical part of the Unit, and one Dirty Utility Room for the COVID-19 section of the Unit. Unit leadership also decided that they would not use the permanent markers for laundry bag capacity, as this increased complexity and workload for staff, as well as to not double-bag linen. In addition, Unit leadership also chose to maintain the use of a single dirty laundry cart in each patient’s room. It was assumed that having a second bag would not discourage staff from overfilling bags with linens. Housekeeping management conducted a staff training session on the Unit the morning after the simulation, to ensure that all the housekeeping staff knew about the new protocols.

Encouraging staff adherence to minimize overfilling laundry bags has required continued education and a new protocol involving scheduled emptying of all linen bags at shift changes. In addition, having a doffing station in the Laundry Chute Room was found to be critical to improving the overall processes involved in handling dirty laundry.

## Discussion

The repurposing of hospital in-patient units for COVID-19 care has been studied in a range of settings [4–14]. The present paper contributes a detailed account of the challenges and solutions involved in rapidly converting a general surgical and orthopedic unit to deliver care to COVID-19 patients in a Canadian context. We have highlighted three key challenges that a designated COVID Unit encountered in the course of its conversion and ‘new normal’ activities, describing efforts to declutter spaces, store equipment, and handle linens in ways that optimize safety and minimize transmission risk. In this sense, we have presented Unit 64 as a case study of issues that may require attention as teams in other hospitals find themselves adapting to pandemic patient flows. Beyond drawing focus to these issues, we have described an interdisciplinary approach to supporting rapid adaptive change in hospital operations. Unit 64’s conversion offered the opportunity for a team of on-Unit managers and staff to participate in an extended interdisciplinary collaboration with HF, ethnographic, and IPC experts and researchers.

IPC professionals are accustomed to sharing responsibility with Public Health, Occupational Health and Safety, and frontline facility staff in managing not just pandemics, but lesser outbreaks in hospitals.

Extending this standard model of interdisciplinary work, a growing body of literature highlights the synergy and collaborative potential between IPC and HF experts [24,29–33]. This work suggests that integrating HF perspectives and skills with IPC can ensure safety protocols balance their focus on both the ‘system’ and ‘the person’ [22]. Epidemic preparedness in acute care has previously benefitted from the integration of HF, for example, by providing key insights during large scale simulations of PPE usage during the response to Ebola [34]. As noted, blending IPC theory of implementation with HF systems and ethnographic ‘alongsider’ approaches was central to assisting Unit 64 in its rapid conversion. Our team’s efforts illustrate the potential synergies in interdisciplinary management of infectious disease outbreaks in acute care–particularly between HF, ethnography, and IPC [24,29–33]. This not only supports health care workers, who have frequent exposure to COVID-19 patients, but also addresses recent Wellcome Trust and World Health Organization (WHO) calls for greater integration of the social sciences in epidemic preparedness and response [35,36].

Ethnographers generally use concepts from sociology and anthropology to describe their work. Ethnography has been established as a key methodology for understanding the context and culture where organizational changes, including healthcare QI interventions, occur [37]. As with HF, they view the world of IPC [38] and healthcare quality improvement (QI) [39] through a lens of systems in which assemblages of people, objects, spaces, affordances, and limitations interact to produce, or counteract, safety [24,40]. Ethnography has become a methodological mainstay of the science of implementation [37,39,41]. In this way, the integration of applied ethnographic observations was a natural next step in enriching the interdisciplinarity brought to the broader project of converting Unit 64 and supporting their COVID-19 care practices.

Working in concert with IPC individual-level or ‘person-centered’ reminders and education, HF contributed structure and process standardization, as well as simplification recommendations, while ethnography provided real-time ‘alongsider’ feedback on the uptake of those recommendations. Our team and Unit 64 staff identified the following elements as most relevant to the success of our contributions: the urgency of the pandemic-induced operational changes to the hospital; the quality and contextual relevance of our recommendations; our status as credible outsiders, with the validity of HF and ethnography expertise strongly supported by IPC leadership; our ongoing efforts to shift from being credible outsiders to trusted collaborators [15,23]; as well as the uncertain transmission patterns and severity of the SARS-CoV-2 virus.

Due to the clinical urgency introduced by the pandemic, the cognitive walkthroughs, task analyses, and ethnographic observations were necessarily conducted in ‘real-time’. There was no opportunity to conduct more formalized data collection, or use other methodologies (e.g., use of the Safety Risk Assessment Tool [42]. This clinical urgency demanded that our recommendations be tailored specifically to Unit 64 at the FMC, and therefore our findings may not be generalizable to other acute care environments in other healthcare systems.

In the case of our initial intervention to de-clutter PPE, we successfully introduced lasting changes to improve safety. In the case of our intervention to optimize the storage of equipment outside patients’ rooms, we produced recommendations that collaboratively and with compromise were able to balance contextual needs with implementation hurdles and adapt our recommendations accordingly. In the case of our intervention to redesign the management of dirty linens, our team was able to identify gaps between the assumptions that were made as IPC guidelines were developed and the actual practices and working conditions of Unit staff and housekeepers. In each of these examples, our team combined IPC reminders and education with HF systems-focused redesign to achieve process standardization and simplification [34]. Our interdisciplinary team was well-placed to balance these elements in response to the rapid changes in COVID-19 protocols in both the hospital and specifically within Unit 64.

## Conclusion

We have provided three examples of specific interventions aimed at the rapid conversion of a general medical unit for COVID-19-based care. These interventions relied on our team’s interdisciplinary triangulation of IPC, HF, and ethnography. This collaborative approach helped us provide recommendations for safety-focused structure and process improvements, which were co-designed alongside Unit leadership and staff. The combination of collaborative engagement and expertise that our team brought to the work bolstered mutual findings and discoveries, and supported frontline healthcare delivery during the COVID-19 pandemic in Alberta.
